# PP2A-Tws dephosphorylates Map205, is required for Polo localization to microtubules and promotes cytokinesis in *Drosophila*

**DOI:** 10.1186/s13008-024-00141-x

**Published:** 2024-12-28

**Authors:** Marine Guelle, Virginie Emond-Fraser, Vincent Archambault

**Affiliations:** Institute for Research in Immunology and Cancer, Département de biochimie et médecine moléculaire, Université de Montréal, Montreal, Québec Canada

**Keywords:** PP2A-B55, Tws, Map205, Polo, Mitosis, Cytokinesis, Cell cycle, *Drosophila*

## Abstract

**Background:**

Mitosis and cytokinesis are regulated by reversible phosphorylation events controlled by kinases and phosphatases. *Drosophila* Polo kinase, like its human ortholog PLK1, plays several roles in this process. Multiple mechanisms contribute to regulate Polo/PLK1 activity, localization and interactions. We previously showed that the microtubule-associated protein Map205 interacts with Polo during interphase and cytokinesis, inhibiting and sequestering Polo on microtubules. During mitosis, phosphorylation of Map205 at a Cyclin-Dependent Kinase site allows Polo to dissociate from Map205, when Polo must fulfill its mitotic functions. How the Polo/Map205 interaction is restored during mitotic exit remained unknown.

**Results:**

Here we show that PP2A-Tws/B55 is required to dephosphorylate Map205, and enables the Map205-dependent localization of Polo to microtubules during cytokinesis. In addition, we show that PP2A-Tws is required for spindle function during cytokinesis, consistent with the essential role of Polo in this process.

**Conclusions:**

These findings complement previous studies to provide an understanding of the full cycle of Polo regulation by Map205, kinases and phosphatases. Our findings have implications for the wider network of cell cycle regulatory circuitry.

**Supplementary Information:**

The online version contains supplementary material available at 10.1186/s13008-024-00141-x.

## Background

**Fig. 1:**
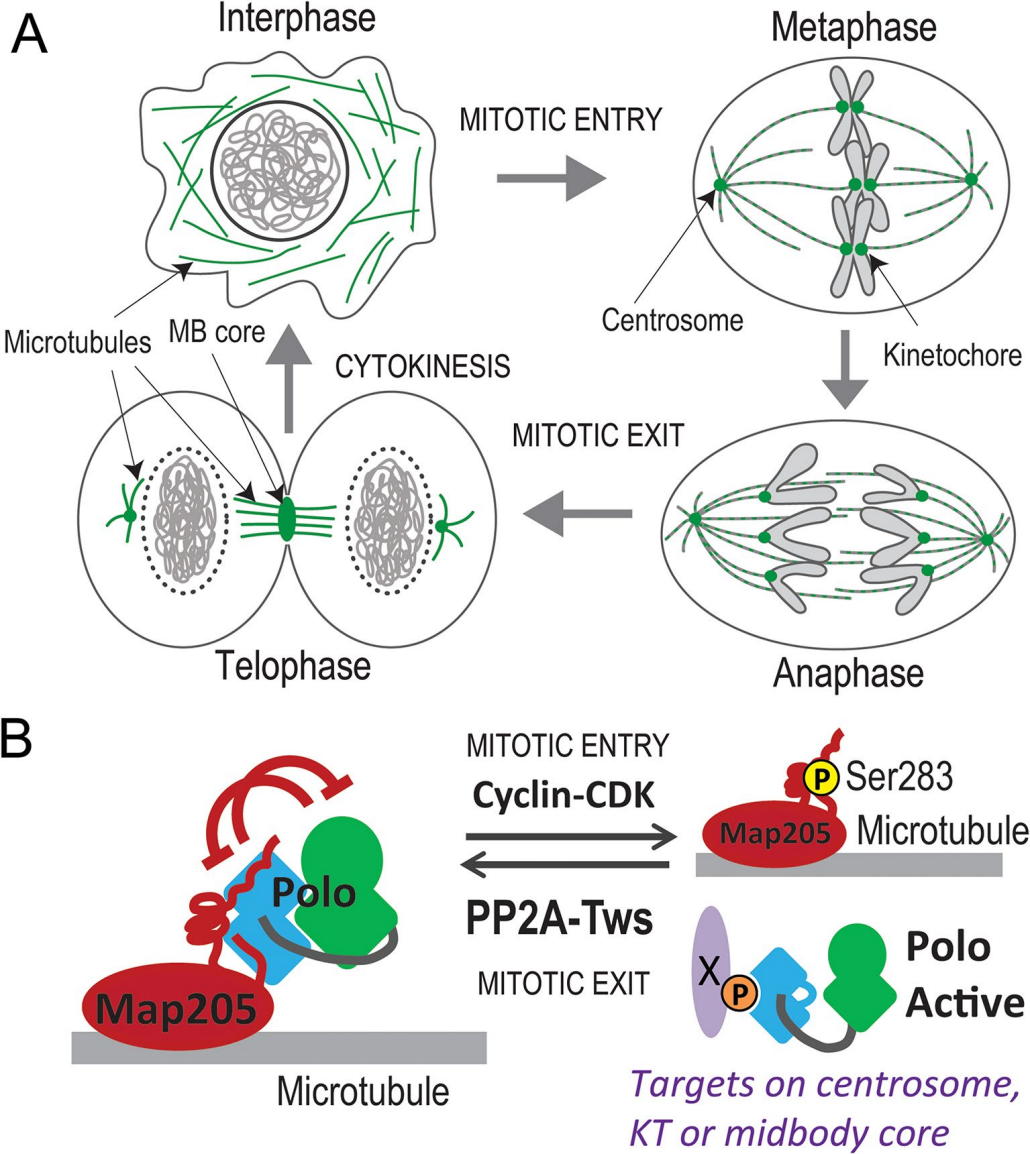
Regulation of Polo localization and function during mitosis and cytokinesis. (**A**) The dynamic localization of Polo during the cell cycle. During mitosis (right), Polo (green) is strongly localized to kinetochores and centrosomes. During cytokinesis and interphase (left), Polo is strongly localized to microtubules. (**B**) Molecular regulation of Polo. During mitosis interphase (left), Polo interacts with Map205 on microtubules. This interaction stabilizes the PBD (blue) of Polo in a conformation that binds and inhibits the KD (green). During mitosis (right), mitotic Cyclin-CDK phosphorylation of Map205 causes the release of Polo. The phosphorylation of Polo interactors (X) facilitates their interactions with the PBD targeting Polo at centrosomes, kinetochores (KT) and the midbody core. During mitotic exit, Map205 dephosphorylation by PP2A-Tws promotes the recruitment of Polo by Map205 on microtubules

The Polo kinase is conserved across many eukaryotes ranging from yeast to vertebrates [1, 2]. Discovered in *Drosophila*, Polo and its closest human ortholog Polo-Like Kinase 1 (PLK1) play essential roles during mitosis and cytokinesis [3, 4]. They comprise a N-terminal Ser/Thr kinase domain (KD) and a C-terminal Polo-Box Domain (PBD) that mediates protein interactions [5, 6]. The PBD binds pre-phosphorylated motifs on proteins of the mitotic centrosomes, centromeres and kinetochores, and of the cytokinetic midbody core, resulting in the localization of Polo/PLK1 at these structures during M-phase [7, 8] (Fig. 1A). At these sites, Polo/PLK1 phosphorylates its interactors or proteins in their vicinity, to modify their functions [5, 9].

In addition, the PBD and KD engage in a reciprocally inhibitory interaction [10, 11]. Phosphorylation of Polo/PLK1 in the T-loop of its KD, or binding of Polo/PLK1 to a pre-phosphorylated target, leads to the dissociation of the two domains, resulting in their activation [10, 12, 13]. Map205 is a microtubule-associated protein that binds Polo, targeting Polo to microtubules (MTs) [14]. Map205 interacts with the PBD of Polo, but in a different manner to other PBD interactors [14]. Rather than inducing the dissociation of the PBD from the KD, Map205 stabilizes the intramolecular interaction, resulting in the inhibition of Polo kinase [13, 15] (Fig. 1B). Although no binding partner has yet been found to inhibit PLK1 by an analogous mechanism in human cells, *Drosophila* Map205 strongly interacts with vertebrate PLK1. The stabilization of the interdomain interaction by Map205 allowed the solving of the first crystal structure of a KD-PBD complex, showing the molecular basis of interdomain inhibition of Polo/PLK1 [15].

We previously showed that when cells enter mitosis, Map205 is phosphorylated at the CDK site Ser283, causing the dissociation of Polo from Map205 when Polo must re-localize to phosphorylate its substrates on kinetochores and centrosomes [14] (Fig. 1B). Phosphorylation of Polo at its T-loop by Aurora B also promotes the dissociation of Polo from Map205, and this regulation is required for the localization of a pool of Polo at the midbody core during cytokinesis [13]. Nevertheless, the Map205-dependent localization of Polo to MTs intensifies during mitotic exit, becoming very strong during cytokinesis. The trigger of this re-localization of Polo is unknown, and whether it simply inhibits Polo or also contributes to cytokinesis is unclear.

Protein Phosphatase 2 A (PP2A) is a Ser/Thr phosphoprotein phosphatase that regulates mitosis [16]. It is composed of a structural subunit (A), a catalytic subunit (C) and a regulatory subunit (B) [17]. Several regulatory subunits can bind PP2A-A in a mutually exclusive manner and mediate substrate specificity and distinct localization patterns to PP2A holoenzymes. B55 subunits confer to PP2A a preference for sites phosphorylated by Cyclin-Dependent Kinases (CDKs), which are followed by a Proline residue [18–23]. As cells enter mitosis, PP2A-B55 is inhibited while mitotic CDKs become active [19, 23]. The mechanism of PP2A-B55 inhibition starts with the activation of Greatwall (Gwl) kinase by its phosphorylation at a CDK site [24]. Gwl then phosphorylates Endosulfines (Ensa and Arpp19 in humans), triggering their selective binding and inhibition of PP2A-B55 [25, 26]. PP2A-B55 ultimately dephosphorylates endosulfines, relieving its own inhibition [27]. PP2A-B55 then becomes able to dephosphorylate other substrates to promote late events of mitotic progression [27, 28]. While PP2A-B55 has been shown to be required for mitotic exit to different degrees in different systems, few of its substrates have been identified [23, 29–34].

The mechanisms described above are conserved in *Drosophila*, where Twins (Tws) is the only B55 subunit, and Endos is the only endosulfine [35, 36]. We previously conducted a proteomic study to identified PP2A-Tws interactors and substrates in *Drosophila* [37]. In this work, we focused on the regulation of Emerin/Otefin by PP2A-Tws during the reformation of the nuclear envelope after mitosis. Among other substrates and interactors, we also identified Map205. Here, we show that Map205 is dephosphorylated by PP2A-Tws to restore its ability to regulate Polo during cytokinesis.

## Results

### PP2A-Tws dephosphorylates Map205 at Ser283

**Fig. 2:**
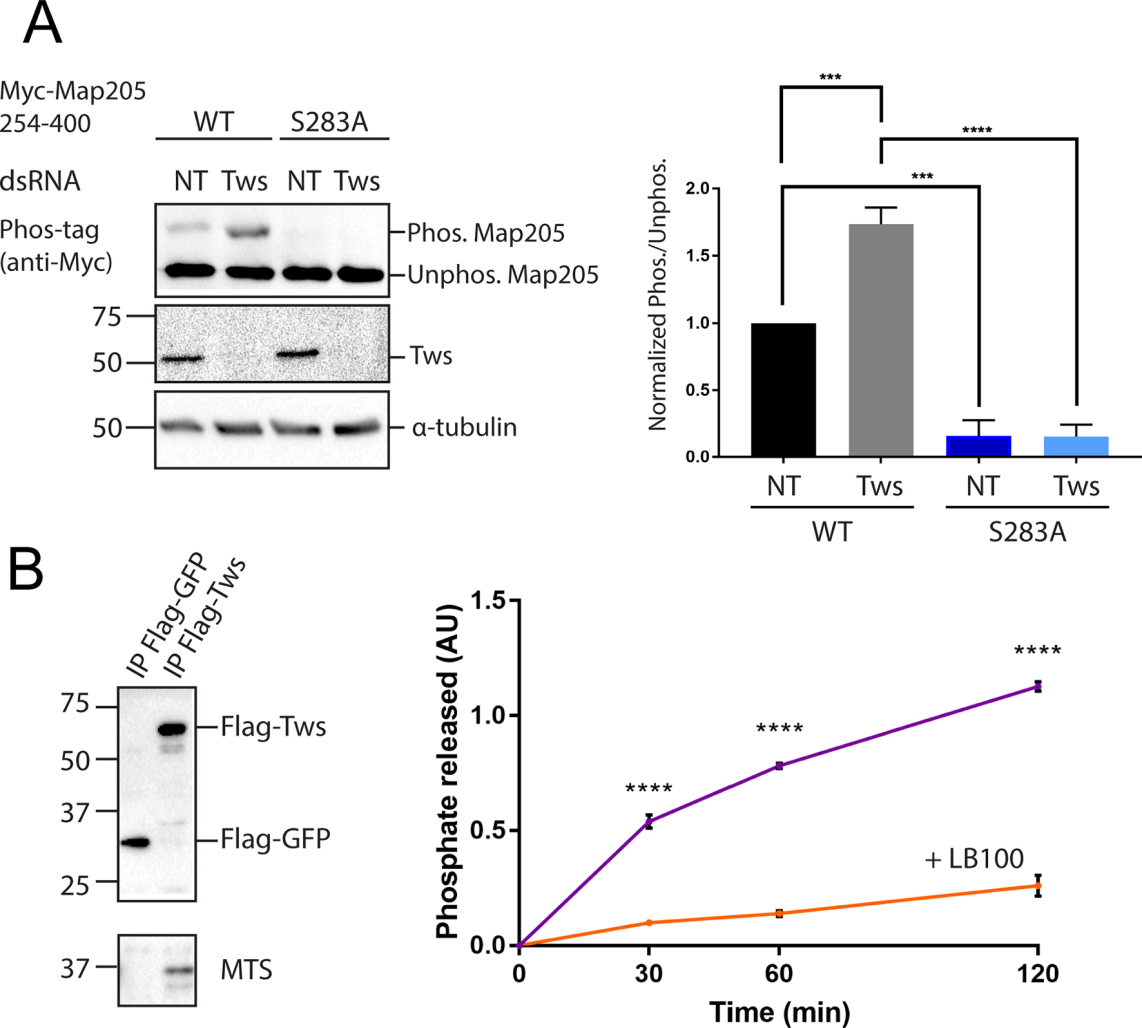
PP2A-Tws dephosphorylates Map205 at Ser283. (**A**) Map205 is hyperphosphorylated at Ser283 after depletion of RNAi depletion of Tws. Cells expressing Myc-Map205_254 − 400_ WT or S283A were transfected with dsRNA against Tws or Non-Target (NT). After 4 days, cells were analyzed by Western blotting using Phos-tag as indicated. Left: results from a representative experiment. Right: Normalized ratios of phosphorylated/unphosphorylated Myc-Map205_254 − 400_ band intensities. Averages of 3 experiments are shown. All error bars: S.D. *** *p* < 0.001, **** *p* < 0.0001 from paired t-tests. (**B**) PP2A-Tws dephosphorylates a Map205 peptide at Ser283 in vitro. Left: Flag-Tws or Flag-GFP were immunoprecipitated from cells and products were analyzed by Western blotting. Right: Dephosphorylation of the pSer283 Map205 peptide by Flag-Tws as a function of time (violet curve). Values obtained with Flag-GFP were subtracted. The Flag-Tws associated phosphatase activity is largely abrogated by the addition of LB100, a PP2A inhibitor (orange curve). Experiment repeated 3 times. Averages of technical triplicates from a representative experiment are shown. AU: arbitrary units. **** *p* < 0.0001 from unpaired t-tests

We previously published a proteomic study of PP2A-Tws that identified Otefin/Emerin as an interactor and substrate whose regulation by PP2A-Tws is required for nuclear envelope reassembly after mitosis [37]. The same study identified Map205 as an interactor of PP2A-Tws in embryos, and found that Map205 became hyperphosphorylated at Ser283 when Tws was depleted in *Drosophila* D-Mel cells in culture. We sought to investigate the regulation of Map205 by PP2A-Tws. To visualize Map205 phosphorylation at Ser283, we used the Phos-tag reagent, which increases electrophoretic mobility upshifts of phosphorylated proteins. Western blot for a fragment of Map205 (amino-acid residues 254–400) tagged with Myc (Myc-Map205_254 − 400_) using D-Mel cell extract revealed two bands (Fig. 2A). Mutation of Ser283 into a non-phosphorylatable Ala residue (S283A) completely abolished the upper band, indicating that this upper band was due to phosphorylation of Myc-Map205_254 − 400_ at Ser283. To test whether this phosphorylation state is regulated by PP2A-Tws, we depleted Tws by RNAi. We found that the phosphorylation of Myc-Map205_254 − 400_ at Ser283 increased approximately two-fold in the Tws-depleted sample compared to a non-target control.

To test if PP2A-Tws directly dephosphorylates Map205 at pSer283, we conducted an in vitro phosphatase assay (Fig. 2B). Flag-Tws was immunoprecipitated from a stable D-Mel cell line. Western blot confirmed the co-purification of Mts, the catalytic subunit of PP2A. As a control, Flag-GFP was immunoprecipitated. Both immunoprecipitation (IP) products on beads were incubated with a Map205 peptide phosphorylated at Ser283. Phosphate released was measured in a colorimetric assay using malachite green. We found that the Flag-Tws IP product could dephosphorylate pSer283 in the Map205 peptide. Phosphatase activity was largely abrogated by the addition of LB100, a PP2A inhibitor [38]. We conclude that PP2A-Tws dephosphorylates Map205 at Ser283.

### PP2A-Tws promotes the localization of Polo to microtubules

**Fig. 3:**
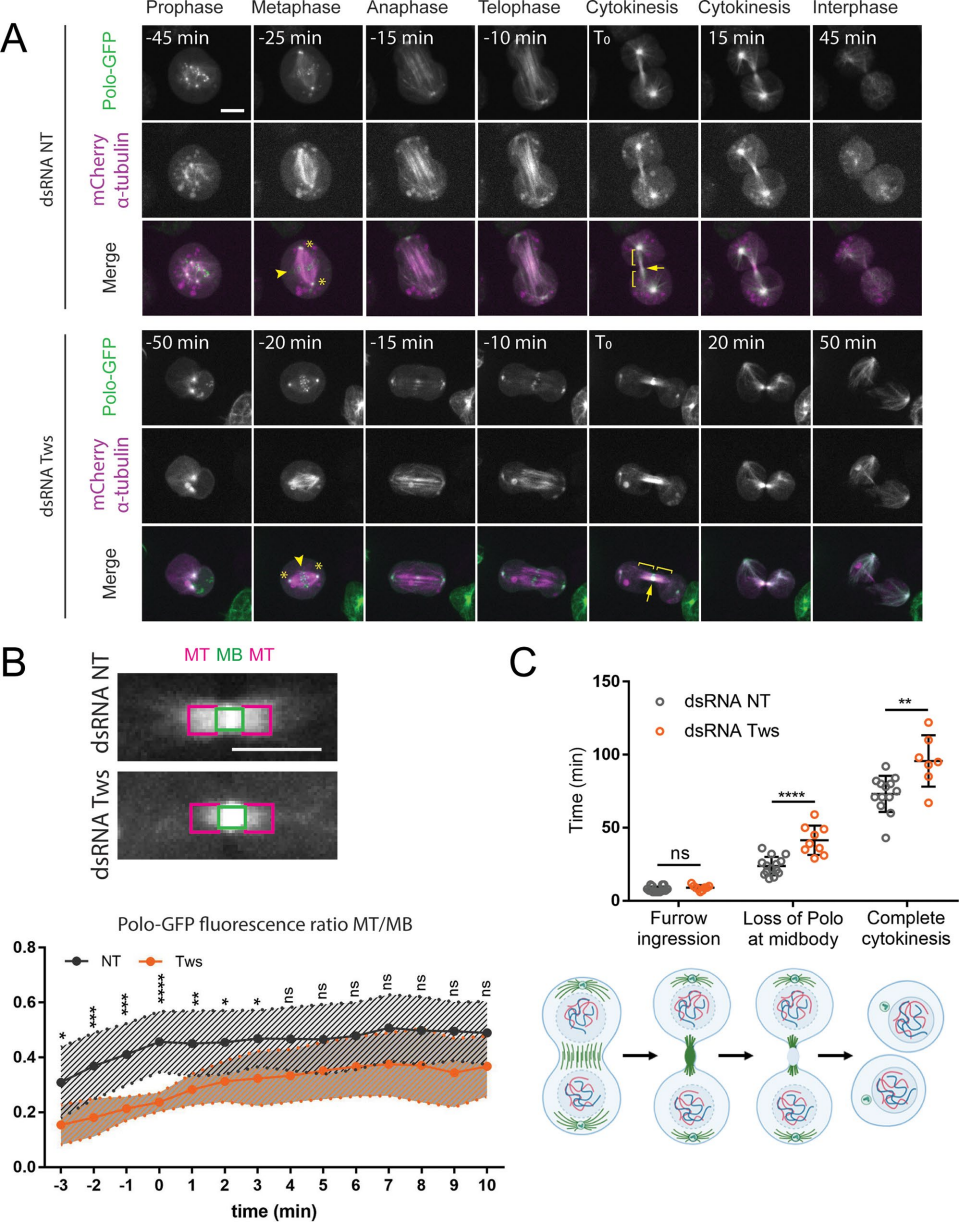
PP2A-Tws promotes the timely localization of Polo to microtubules and timely abscission during cytokinesis in D-Mel cells. (**A**) Cells expressing Polo-GFP and mCherry-Tubulin were filmed during their divisions. In control cells (RNAi NT, top), Polo-GFP becomes strongly localized to microtubules during telophase and cytokinesis. By contrast, in Tws-depleted cells (RNAi Tws, bottom), Polo-GFP is less strongly localized to microtubules (backets). However, the localization of Polo-GFP to centrosomes (asterisks), kinetochores (arrowheads) and the recently constricted midbody core (arrow) are not affected. Scale bar: 5 μm. (**B**) Quantification of Polo-GFP localization to microtubules relative to the midbody core during cytokinesis. Top: GFP fluorescence intensity measurements were taken on central spindle microtubules (MT, magenta boxes) and at the midbody core (MB, green boxes). Scale bar: 5 μm. Bottom: the ratio of MT/MB fluorescence over time was plotted as a function of time. T_0_: end of furrow ingression. Between 10 and 20 cells were quantified per condition. All error bars: S.D. * *p* < 0.05, **p < 0.01, ***<0.001, **** *p* < 0.0001, ns: non-significant from two-way Anova. (**C**) Quantifications of the durations of furrow ingression, retention of Polo-GFP at the midbody core and complete cytokinesis (until abscission) relative to anaphase onset in D-Mel cells depleted of Tws or control cells. The cartoons at the bottom illustrate the stages quantified. Between 7 and 13 cells were quantified in each condition. All error bars: S.D. * *p* < 0.05, **p < 0.01, ***<0.001, **** *p* < 0.0001, ns: non-significant from unpaired t-tests

We previously showed that phosphorylation of Map205 at Ser283 disrupts its interaction with Polo [14]. In addition, the localization of Polo on MTs depends on Map205 [13, 14]. Thus, we wondered if PP2A-Tws, by dephosphorylating Map205, promotes the localization of Polo to MTs. To test it, we imaged mitosis in D-Mel cells expressing Polo-GFP. We observed the localization of Polo-GFP at centrosomes and kinetochores during mitosis, at the cytokinetic midbody core and at MTs, as previously reported (Fig. 3A, top). We previously showed that only the localization of Polo at MTs depends on Map205 [14], while Polo localization at centrosomes, kinetochores and the midbody core depends on other interactions [1]. In particular, the intense localization of Polo at the midbody core, the focal structure at the site of cytokinesis, depends on Fascetto (Feo), the ortholog of human PRC1 [39]. We found that upon depletion of Tws by RNAi, the localization of Polo-GFP at MTs was strongly decreased, while other localizations of Polo-GFP were not noticeably perturbed (Fig. 3A, bottom). To quantify this effect, we measured the ratio of Polo-GFP fluorescence at central spindle MTs relative to Polo-GFP fluorescence at the midbody core through time, taking the time of the end of furrow ingression as T_0_ (Fig. 3B). We found that the intensity of Polo-GFP at MTs was strongly reduced during and after furrow ingression.

**Fig. 4:**
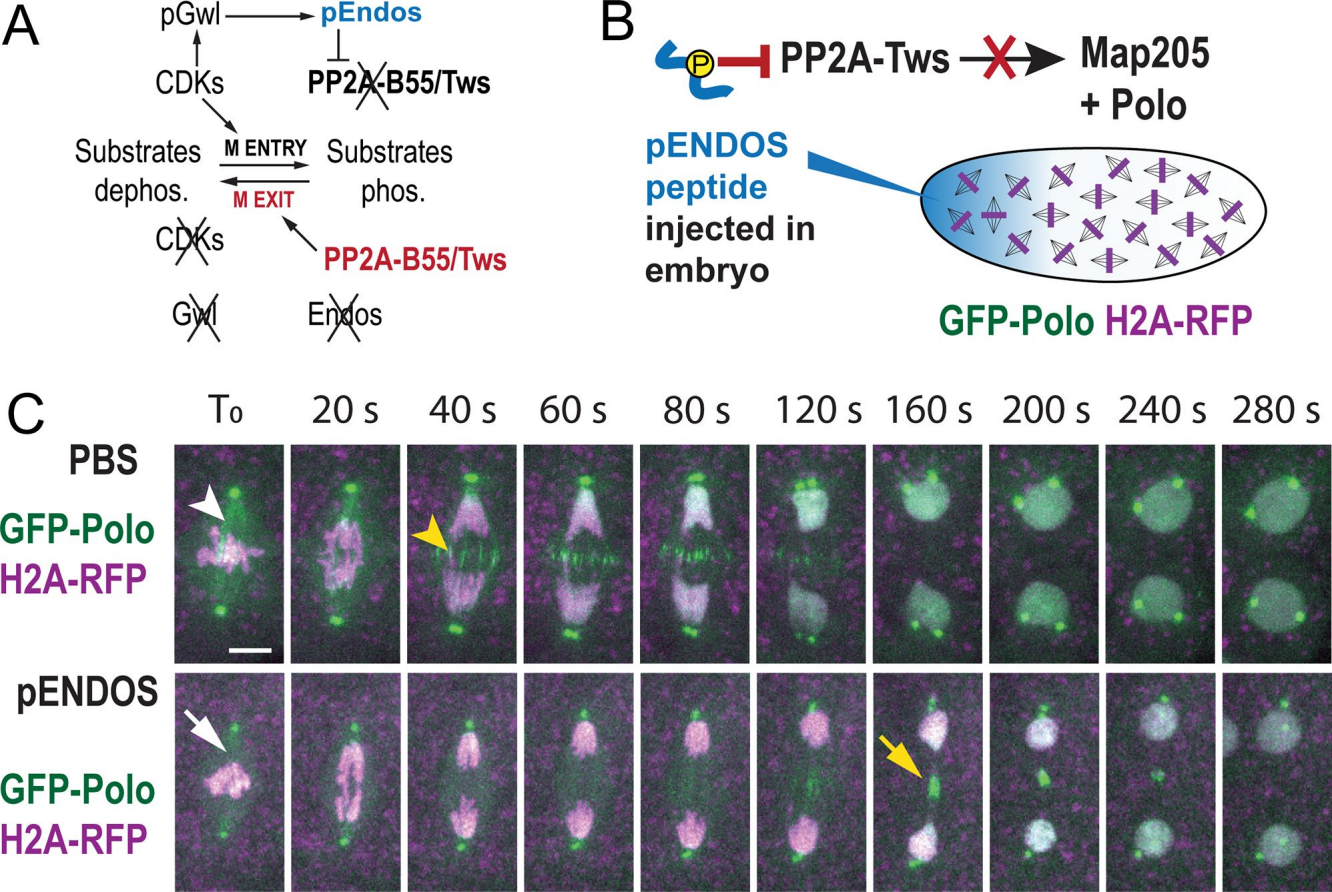
PP2A-Tws promotes the localization of Polo to microtubules during karyokinesis in syncytial embryos. (**A**) PP2A-B55/Tws is inhibited by phosphorylated Endos during mitotic entry. During mitotic exit, PP2A-B55/Tws is reactivated when Endos is dephosphorylated. See text for more details. **B-C**. Injection of a phosphopeptide from Endos (pENDOS) to inhibit PP2A-Tws causes the mislocalization of GFP-Polo-GFP to the central spindle during karyokinesis. (**B**) Experimental scheme. (**C**) Transgenic embryos expressing GFP-Polo and H2A-RFP were imaged immediately after injection. T_0_: immediately before anaphase onset. After control injection (PBS only, top) during metaphase, GFP-Polo is faintly localized to microtubules already in metaphase (white arrowhead) and becomes concentrated on the central spindle after anaphase (yellow arrowhead). After injection of the pENDOS phospho-peptide, the localization of GFP-Polo to microtubules is strongly reduced (white arrow). In addition, GFP-Polo accumulates on the central spindle later, at the midbody core (yellow arrow). Scale bar: 5 μm

To test if the localization of Polo depends on PP2A-Tws in vivo, we used syncytial *Drosophila* embryos expressing GFP-Polo and Histone 2 A-RFP (H2A-RFP, which marks chromosomes). *tws* is an essential gene [40, 41] and embryos where Tws is inactivated by RNAi fail to develop (our unpublished observations). In addition, no selective chemical inhibitor of PP2A-B55/Tws is currently available. To circumvent these hurdles, we injected pENDOS, a phospho-peptide that selectively inhibit PP2A-Tws. The peptide is derived from Endos (endosulfine), which becomes a PP2A-Tws inhibitor when phosphorylated by Greatwall during mitotic entry [25, 26, 35] (Fig. 4A-B). We recently showed that injection of the pENDOS peptide in syncytial embryos results in a delay in nuclear envelope reformation that is consistent with the role of PP2A-Tws in dephosphorylating Otefin and BAF to promote this process [37]. We found that injection of the pENDOS peptide in syncytial embryos abrogated the localization of GFP-Polo at MTs (Fig. 4C). In PBS-injected, control embryos, GFP-Polo was already weakly localized to MTs during metaphase. After anaphase, GFP-Polo was clearly localized to MTs of the central spindle, while karyokinesis was quickly completed. By contrast, upon injection of the pENDOS peptide, GFP-Polo was delocalized from MTs from metaphase to telophase. After a long delay compared to control embryos, GFP-Polo became concentrated on the constricted midbody cores of persisting karyokinetic figures. Nuclear expansion was also delayed by the pENDOS peptide, consistent with the role of PP2A-Tws in nuclear envelope reassembly [37, 42]. In pENDOS-injected embyos, GFP-Polo was retained on the midbody much later that in control embryos. Consistent results were obtained in D-Mel cells where depletion of Tws also resulted in a longer retention of Polo-GFP at the midbody core (Fig. 3C). Altogether, we conclude that PP2A-Tws promotes the timely localization of Polo to spindle MTs and the midbody core during M-phase.

### PP2A-Tws is required for central spindle dynamics during cytokinesis

**Fig. 5:**
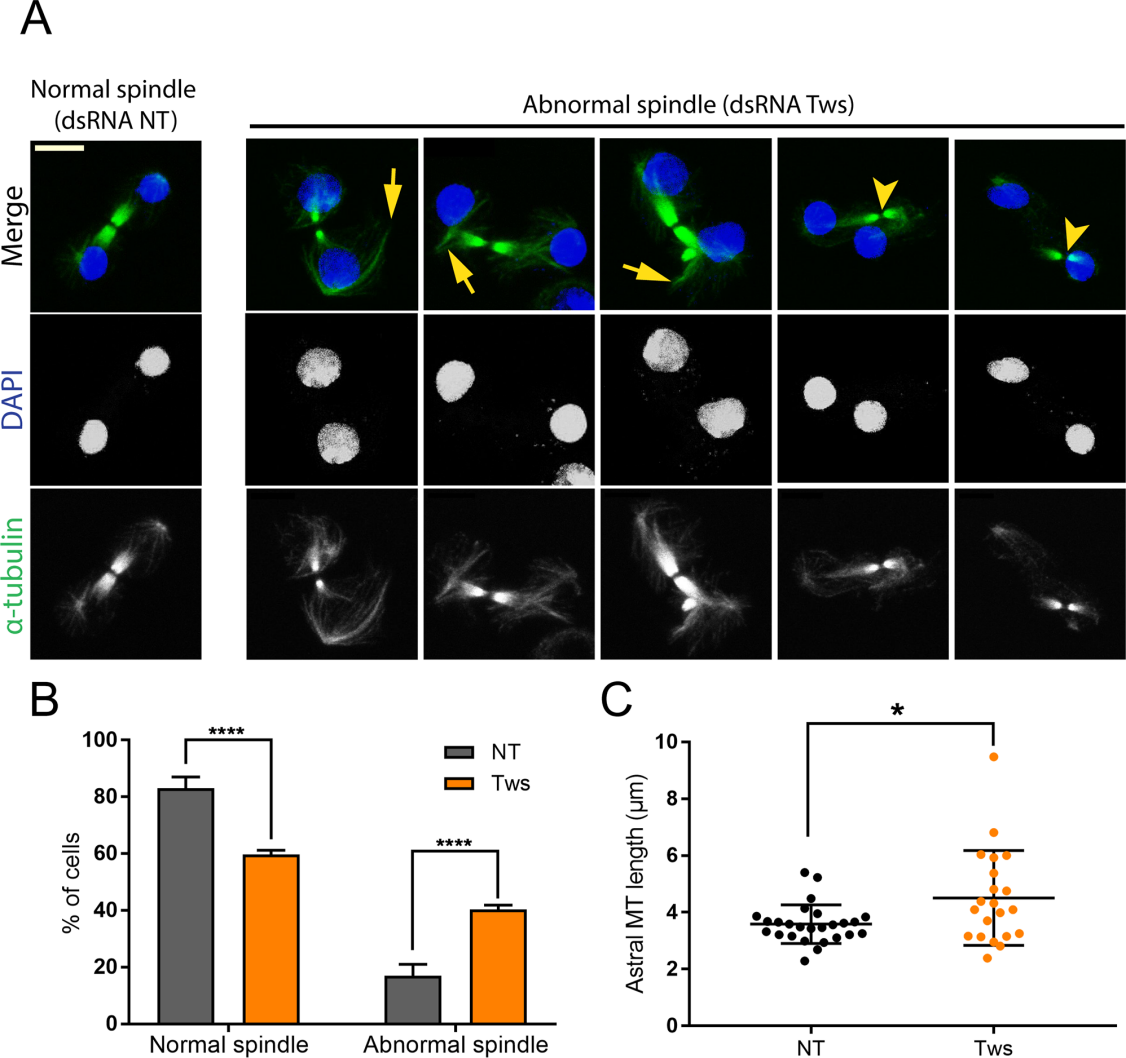
PP2A-Tws is required for spindle function during cytokinesis. (**A**) D-Mel cells were transfected with dsRNA against Tws or non-target (NT). After 3 days, cells were analyzed by immunofluorescence. The depletion of Tws results in cells with abnormal spindles showing bundles of microtubules that are distinct from or oriented away from the main spindle (arrows). Spindles are also mispositioned relative to daughter nuclei (arrowheads). (**B**) Quantification of abnormal vs. normal spindles from experiments as in A. Averages from 3 experiments are show. 100 cells were quantified per condition in each experiment. All error bars: S.D. **** *p* < 0.0001 from paired t-tests. (**C**) Quantification of maximal astral microtubule lengths from experiments as in A. Twenty-six (NT) and 21 (Tws) cells were quantified. * *p* = 0.02 from paired t-test

Polo kinase activity is required for cytokinesis [43–48]. We previously showed that the regulation of Polo activity and localization by Aurora B kinase and Map205 promotes this role [13]. Since PP2A-Tws dephosphorylates Map205 and promotes the recruitment of Polo to midbody MTs, we tested if PP2A-Tws is required for normal cytokinesis. To this end, we examined Tws-depleted cells by immunofluorescence. We found that Tws RNAi resulted in abnormal spindles, which were frequently splayed. Separate bundles of MTs were oriented away from the central spindle which was often mispositioned relative to daughter nuclei (Fig. 5A-B). Asters were also longer after Tws depletion (Fig. 5C). In addition, we observed an extension of the total duration of cytokinesis after depletion of Tws (Fig. 3C). We conclude that the action of PP2A-Tws is required for the proper function of the central spindle and for timely completion of cytokinesis.

## Discussion

From previous work, it was known that Map205 interacts with Polo, sequesters Polo to MTs and inhibits Polo activity in interphase [13–15]. It was known that phosphorylation of Map205 at a CDK site (Ser283) disrupts its interaction with Polo, consistent with the relocalization of Polo to centrosomes and kinetochores, where Polo activity is required in mitosis [14]. Here, we present several lines of evidence that suggest that the Map205-dependent localization of Polo to MTs is restored by dephosphorylation of Map205 at Ser283 by PP2A-Tws during mitotic exit, completing the regulatory circuit in the entire cell cycle (Fig. 1). Interestingly, while Polo localization to MTs increases during mitotic exit, it is already visible on the spindle during early mitosis, and this localization is also dependent on PP2A-Tws. This suggests that the Polo-Map205 interaction is subject to a shifting equilibrium that depends on PP2A-Tws throughout the cell cycle.

We also found that depletion of Tws results in central spindle defects and cytokinesis delays. Because Polo is required for cytokinesis [1, 3, 45], we propose that PP2A-Tws could promote cytokinesis in part by regulating Polo, although we are aware that the link is correlative and no causality has been formally demonstrated. While Map205 inhibits Polo, we previously showed that the complex also stabilizes Polo [14]. We previously showed that the activating phosphorylation of Polo by Aurora B promotes the dissociation of Polo from Map205 by an allosteric mechanism, and the re-localization of a pool of Polo from the central spindle MTs to the cytokinetic midbody core. Failure of this regulation leads to cytokinetic defects [13]. Thus, the dephosphorylation of Map205 by PP2A-Tws may not only result in the inactivation of Polo on MTs, but also in the stabilization of Polo in a pool that can be mobilized by Aurora B for Polo recruitment and function at the midbody core. This dual regulation could reconcile apparently contradictory genetic interactions results. On the one hand, overexpression of Map205 enhanced the lethality of embryos from *polo*^*−/+*^ mothers in a manner that depends on the ability of Map205 to interact with Polo (Map205 S283A was more toxic, while Map205 S283D was less toxic than Map205 WT), consistent with Map205 inhibiting Polo [14]. On the other hand, mutation of one allele of *tws* also enhanced the lethality of these embryos (embryos from *polo*^*−/+*^, *tws*^*−/+*^ mothers failed to hatch) [36]. This observation suggests a collaboration between PP2A-Tws and Polo that could be mediated by the stabilization of Polo through its interaction with Map205.

In humans as in *Drosophila*, PP2A-B55/Tws promotes various events of mitotic exit and cytokinesis [32, 49]. Our previous work in *Drosophila* demonstrated that PP2A-Tws promotes the reassembly of the nucleus by dephosphorylating BAF and Otefin [37, 42]. Here, we characterized the regulation of an additional substrate of PP2A-Tws, Map205, that emerged from our recently published phosphoproteomic analysis [37]. While the function of Map205 in regulating Polo appears not to be strictly conserved in humans, PP2A-B55 may be connected to PLK1 by alternative mechanisms achieving similar outcomes. In particular, the timing of recruitment of PLK1 by the MT-associated protein PRC1 on the cytokinetic central spindle has been shown to be dictated by the dephosphorylation of CDK1 sites on PRC1 by PP2A-B55 [28]. In *Drosophila*, while Polo recruitment to the spindle midzone that becomes the midbody core during cytokinesis depends on Feo [39], protein sequence comparison between Feo and its human ortholog PCR1 suggests that the phospho-regulation of a putative interaction of Feo with Polo is unlikely to be conserved. Overall, the circuitry regulating cytokinesis and connecting CDK1, PP2A-B55/Tws and Polo/PLK1 is another illustration of the importance of cross-talk between kinases and phosphatases in cell division, and of the evolutionary plasticity in its specific mechanisms.

## Materials & methods

### Plasmids and mutagenesis

Expression vectors for *Drosophila* cells were generated by Gateway recombination (Invitrogen). The coding sequences of the genes were cloned into the entry vector pDONR221 and then recombined into either copper-inducible (pMT) or constitutive (pAC5) expression vectors. Site-directed mutagenesis was done using the QuickChange II kit (Agilent). The following expression vectors were used: pMT-Polo-GFP, pMT-Myc-Map205^254–400^, pMT-Myc-Map205^254–400 S283A^, pAC5-Flag-GFP, pAC5-Flag-Tws, and pAC5-mCherry-α-tubulin.

### Cell culture

All cells were in the D-Mel (D-Mel2) background and were cultured in Express Five (Invitrogen) or EX-CELL 420 (Sigma) medium supplemented with glutamine, penicillin, and streptomycin (Wisent). Transfections were performed using X-tremeGENE HP DNA Transfection Reagent (Roche) following the manufacturer’s instructions. All stable cell lines were selected in medium containing 20 µg/ml blasticidin. While inducible pMT vectors contained the blasticidin resistance gene, pAc5 vectors were co-transfected with pCoBlast to confer blasticidin resistance to the cells. Expression of inducible transgenes was induced with CuSO_4_ (300 µM) for at least 8 h.

For RNA interference experiments, the dsRNAs were produced from PCR amplicons using the T7 RiboMAX kit (Promega) following the manufacturer’s instructions. As a non-target control, a dsRNA directed against the bacterial kanamycin resistance gene was used. Twenty µg of dsRNA were added to 2 million cells in 2 mL of medium in a well of a 6-well plate. Oligonucleotide sequences were:

T7-Tws-F: TAATACGACTCACTATAGGGAGATCCTGCCTCAAAAGCC.

T7-Tws-R: TAATACGACTCACTATAGGGAGAGAAGGTCTCCTGATCCGA.

T7-Kan-F: TAATACGACTCACTATAGGGAGACGACAATCTATCGCTTGTATGG.

T7-Kan-R: TAATACGACTCACTATAGGGAGACCGTCAGCCAGTTTAGTCTG.

### Western blots

For standard SDS-PAGE, cells were collected and washed in PBS containing protease inhibitors (1 mM PMSF, 10 µg/mL Aprotinin, and 10 µg/mL Leupeptin). For SDS-PAGE using Phos-tag, Cells were washed with TBS (50 mM Tris, 150 mM NaCl pH 7). Cells were then lysed in Laemmli buffer at 95 °C for 5 min. Samples were then run on an SDS-PAGE gel and proteins were transferred onto PVDF. Membranes were incubated for 45 min in blocking solution (PBS with 0.1% Tween 20 and 5% dry milk). Antibodies were diluted in blocking solution. Primary antibodies were incubated for at least 2 h and the secondary antibodies for 45 min. Three 10-minute washes with PBS + 0.1% Tween 20 were done after incubations with the primary and secondary antibodies. Membranes were then incubated with Clarity Western ECL Substrate (#170–5061, Bio-Rad) and imaged using the ChemiDoc™ MP system. To evaluate the phosphorylation level of the protein of interest, protein lysates were separated by SDS-PAGE in the presence of Phos-tag (Fujifilm WAKO Chemical), following the manufacturer’s instructions. For this experiment, the PBS was replaced by TBS. Also, one wash of the acrylamide gel with the transfer buffer containing 1 mM EDTA and a second wash with only the transfer buffer were performed before the transfer on a membrane.

The primary antibodies used were anti-Myc 9E10 from mouse (#sc-40 Santa Cruz Biotechnology, at 1:1000), anti-Flag M2 from mouse (#F1804 Sigma-Aldrich, at 1:2000), anti-Tws from rabbit (custom made by Thermo Fisher Scientific, at 1:1000), anti-PP2A B subunit from rabbit (#2290P New England Biolabs, at 1:1000), and anti-tubulin DM1A from mouse (#T6199 Sigma-Aldrich, at 1:5000). Secondary anti-rabbit and anti-mouse antibodies coupled with peroxidase were used (Jackson ImmunoResearch, 1:5000).

Uncropped images of Western blots are provided as a supplementary file.

### Immunofluorescence

Cells were fixed for 15 min at room temperature with a solution containing 4% formaldehyde, 60 mM PIPES at pH 6.8, 30 mM HEPES at pH 7, 10 mM EGTA at pH 6.8, and 0.4 mM MgSO₄. After 2 × 5 min washes with PBS, cells were permeabilized and blocked with a PBS solution containing 0.1% Triton X-100 and 1% BSA (PBSTB) for 1 h. The primary antibodies were diluted in PBSTB and incubated for 2 h at room temperature. After 3 × 5-minute washes with PBSTB, cells were incubated with the secondary antibody diluted in PBSTB and DAPI for 2 h at room temperature in the dark and washed twice for 10 min with PBS. The primary antibody anti-tubulin DM1A from mouse (#T6199 Sigma-Aldrich at 1:2000) and the secondary antibody anti-mouse conjugated to Alexa-488 (Invitrogen, 1:200) were used.

### Phosphatase assay

Approximately 200 million D-Mel cells stably expressing Flag-Tws or Flag-GFP were collected and centrifuged at 1,500 rpm for 5 min at 4 °C. Pellets were resuspended in TBS containing protease inhibitors (1 mM PMSF, 10 µg/ml aprotinin, and 10 µg/ml leupeptin). Cells were lysed in buffer containing 20 mM Tris-HCl, pH 7.5, 150 mM NaCl, 2 mM EGTA, 0.5% NP-40, 1 mM DTT, and protease inhibitors (as above) and incubated on a wheel for 15 min at 4 °C before being centrifuged at 4,600 rpm for 15 min at 4 °C. Supernatants were incubated with anti-Flag antibody (dilution 1:400) for 1 h 30 min on a wheel at 4 °C and with protein G–conjugated Dynabeads (Life Technologies) for an additional 45 min. Beads were washed 4 × 5 min with lysis buffer before incubation with supernatants. Beads were then resuspended in Tris-NaCl solution (Tris 20 mM, NaCl 150 mM) with or without LB100 at 100 µM. All phosphatase assay reactions were done in triplicates. The pSer283 Map205-derived phosphopeptide used as a substrate was GGTLDDLVAE(pS)PRKEFARINM (custom made by Biomatik). Each reaction solutions contained 200 µM peptide, 20 mM Tris, pH 7.5, 5 mM MgCl_2_, 1 mM EGTA, 20 mM β-mercaptoethanol, and 0,1 mg/ml of BSA. Thirty µl of reaction solution and 30 µl of bead suspensions were combined and incubated at room temperature in 96-well plates. Reactions were stopped by the addition of 90 mM HClO_4_. Phosphate released was revealed by the addition of one volume of 1 M malachite green solution. The absorbance was measured at a wavelength of 620 nm using a plate reader (Tecan Infinite 200 PRO). For results presented in Fig. 2B, the colorimetric measurements obtained with Flag-GFP for each time points were subtracted from the measurements obtained with Flag-Tws. Values for T_0_ were also subtracted from both series.

### Live microscopy

Live imaging was performed using a spinning-disc confocal system (Yokogawa CSU-X1 5000) mounted on a fluorescence microscope (Zeiss Axio Observer Z1) using an Axiocam 506 mono camera (Zeiss), 63X oil objective (NA 1.4) and ZEN software. D-Mel cells in culture were plated in a Lab-Tek II chambered coverglass (no. 155409; Thermo Fisher Scientific). Acquisition was performed every 30 s across 7 planes spaced by 1 μm. In Fig. 3B, the average fluorescence intensity was measured using ZEN software.

For live analysis of *Drosophila* syncytial embryos, 0 to 2 h old embryos were first dechorionated in 50% bleach, aligned on a coverslip (no. P35G-1.5-14-C; MatTek) and covered with halocarbon oil. Acquisition was performed every 20 s and planes were spaced by 1 μm. Embryo injections were performed using a home-made glass capillary fixed on an electronically controlled micromanipulator installed on the microscope. Injections were triggered manually using an air-filled plastic syringe connected to the capillary through a rubber tube. The embryos were injected with a 10 mM solution of a PP2A-Tws inhibitory peptide derived from Endos and phosphorylated at its Gwl site (pENDOS: amino acid residues 41–80, pSer67) or with PBS as a control.

### Statistical analysis

GraphPad software was used for making graphs and statistical analyses. All results are expressed as mean ± SD unless otherwise indicated. Sample size and tests used are specified in each figure legend. In all figures, p values are represented as follows: *: *p* < 0.05; **: *p* < 0.01; ***: *p* < 0.001; and ****: *p* < 0.0001, and n.s.: *p* > 0.05. Data distribution was assumed to be normal, but this was not formally tested.

## Electronic supplementary material

Below is the link to the electronic supplementary material.

Supplementary Material 1

## Data Availability

Data is provided within the manuscript.
