# Supplementary material for: PP2A-Tws dephosphorylates Map205, is required for Polo localization to microtubules and promotes cytokinesis in Drosophila
Source: Cell Div. 2024 Dec 28;19:36. doi: 10.1186/s13008-024-00141-x (PMC11682627; doi:10.1186/s13008-024-00141-x)
Supplement: Supplementary file 1 — Supplementary Material 1 [file 13008_2024_141_MOESM1_ESM.pdf]

Uncropped images of Western Blots in Fig.2A

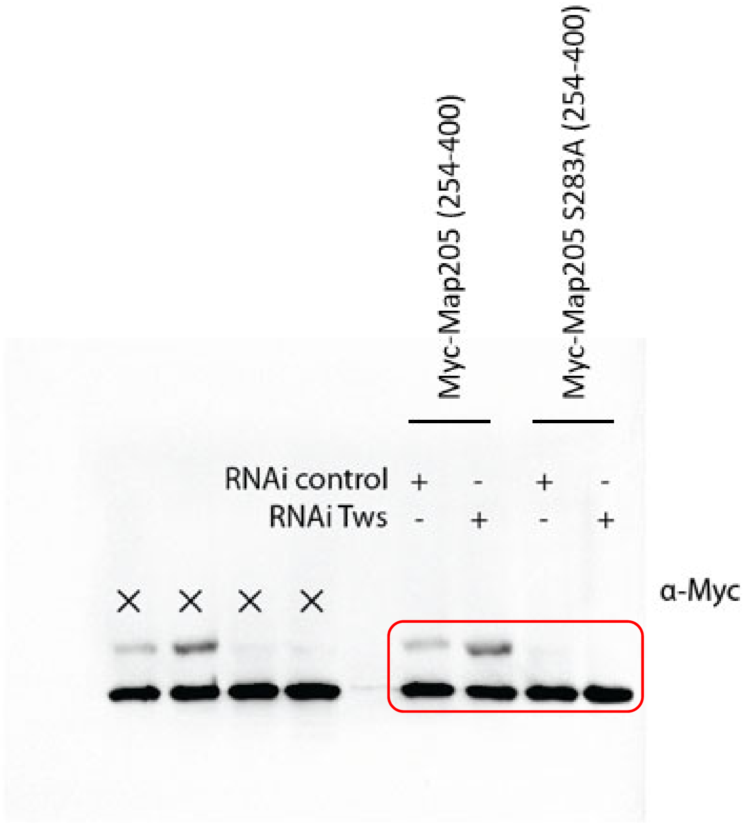

Western Blots were revealed using the Clarity Western ECL substrate kit. Images were taken using a ChemiDoc system. Cropped images shown in Fig 2 are indicated in red rectangles.

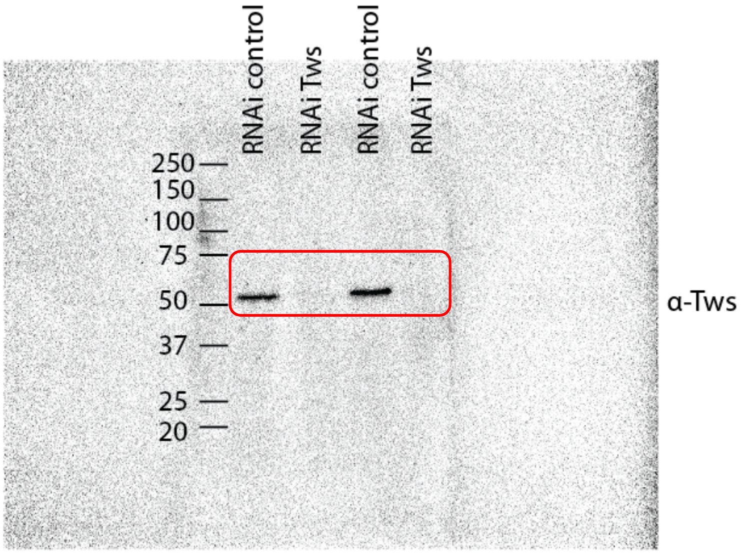

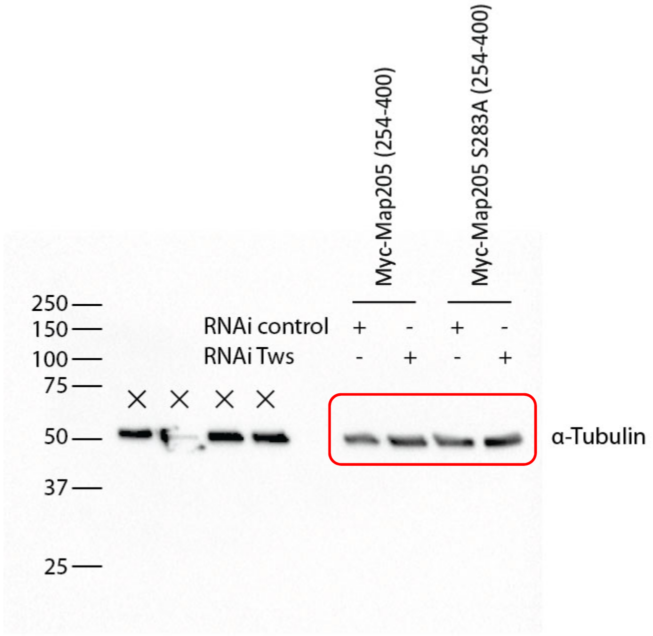

Uncropped images of Western Blots in Fig.2B

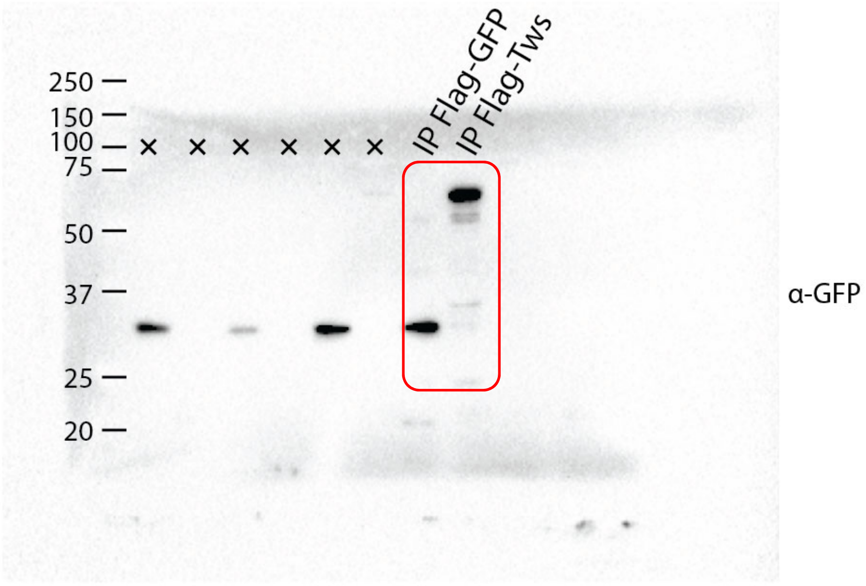

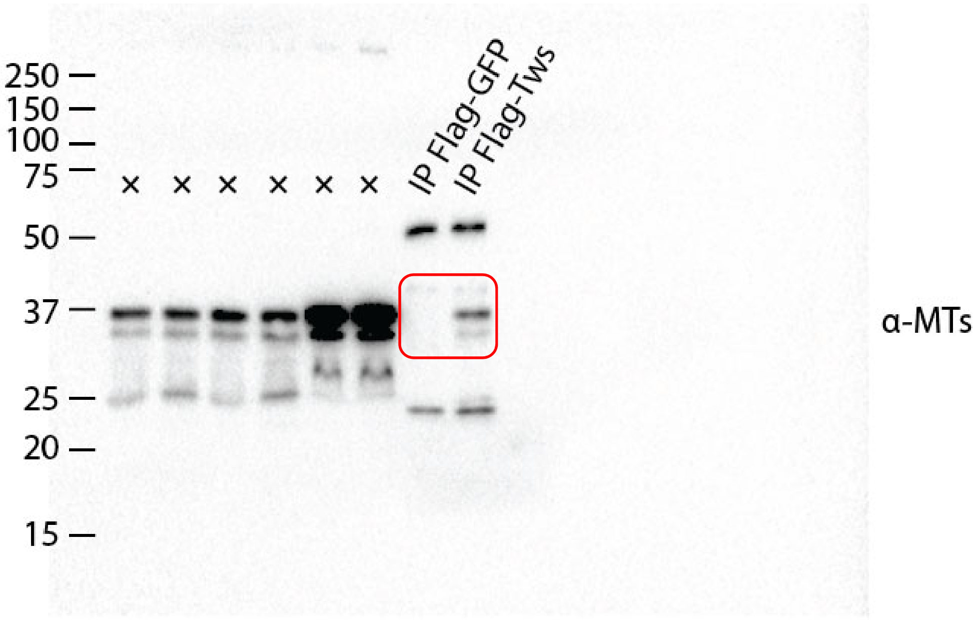

250
150
100
75
50
37
25
20
15
IP Flag-GFP
IP Flag-Tws
α-MTs
